# The Case of Watson vs. James: Effect-Priming Studies Do Not Support Ideomotor Theory

**DOI:** 10.1371/journal.pone.0054094

**Published:** 2013-01-22

**Authors:** Ralf F. A. Cox, Fred Hasselman

**Affiliations:** 1 Heymans Institute, University of Groningen, Groningen, The Netherlands; 2 Behavioural Science Institute, Radboud University Nijmegen, Nijmegen, The Netherlands; University of Sydney, Australia

## Abstract

In this paper we show that response facilitation in choice reaction tasks achieved by priming the (previously perceived) effect is based on stimulus-response associations rather than on response-effect associations. The reduced key-press response time is not accounted for by earlier established couplings between the key-press movement and its subsequent effect, but instead results from couplings between this effect and the contingent key-release movement. This key-release movement is an intrinsic part of the entire performed response action in each trial of a reaction-time task, and always spontaneously follows the key-press movement. Eliminating the key-release movement from the task leads to the disappearance of the response facilitation, which raises the question whether response-effect associations actually play a role in studies that use the effect-priming paradigm. Together the three experiments presented in the paper cast serious doubts on the claim that action-effect couplings are acquired and utilized by the cognitive system in the service of action selection, and that the priming paradigm by itself can provide convincing evidence for this claim. As a corollary, we question whether the related two-step model for the ideomotor principle holds a satisfying explanation for how anticipation of future states guides action planning. The results presented here may have profound implications for priming studies in other disciplines of psychology as well.

## Introduction

William James’ ideomotor principle suggests that anticipation of the consequences of movements is an important, if not essential, feature of action control [1]. In the past few decades a strong case has been built in support of the ideomotor principle and its role in voluntary goal-directed action (e.g. [2]–[4]; for an overview see [5]). By now ideomotor thinking has been widely adopted across various fields of psychology and it is generally believed to be one of the fundamental mechanisms underlying human behavior. Applications can be found in research on social cognition [6]–[9], imitation [10]–[12], as well as in several neuroscience studies [13]–[16]. In addition, several studies on the development of means-end behavior and memory and retention, such as for instance the famous ‘mobile’ conditioning paradigm by Rovee-Collier and coworkers [17], [18] have been reinterpreted to fit the framework of ideomotor theory (see e.g. [19]).

In contemporary theoretical accounts of the ideomotor principle it is argued that the intention of bringing about an external sensory effect (e.g., the sound of a ringing doorbell) triggers the appropriate motor codes that led to the production of the effect at previous occasions (i.e., pushing the button). Moreover, it is claimed that actions are actually arranged this way within the cognitive system, that is, in a common-coding framework [4]. To be more specific, motor codes are represented according to the codes of their associated sensory events. In the present study however, we report evidence suggesting that such simple one-to-one and bidirectional associations between effect representations and action representations insufficiently capture the mechanisms underlying the ideomotor principle.

Models of the mechanisms of ideomotor action control generally consist of two distinct stages (see e.g. [20]). The first stage concerns the formation of couplings between performed movements and their associated consequences (effects) in the environment. In the second stage, voluntary goal-directed action is initiated by anticipating on the effect and using the previously formed association for the recruitment of appropriate (effect-related) motor codes. This two-step model affords a straightforward empirical design for testing its viability [2]: Arbitrary associations between movements (e.g. in response to irrelevant stimuli) and effects should be learnable in a task by repeatedly pairing them in an acquisition phase, such that the former appears to specifically produce the latter. Afterwards, in a separate utilization phase, the mere occurrence of one of the effects (i.e., priming) should trigger the associated action, or at least speed up its selection.

**Figure 1:**
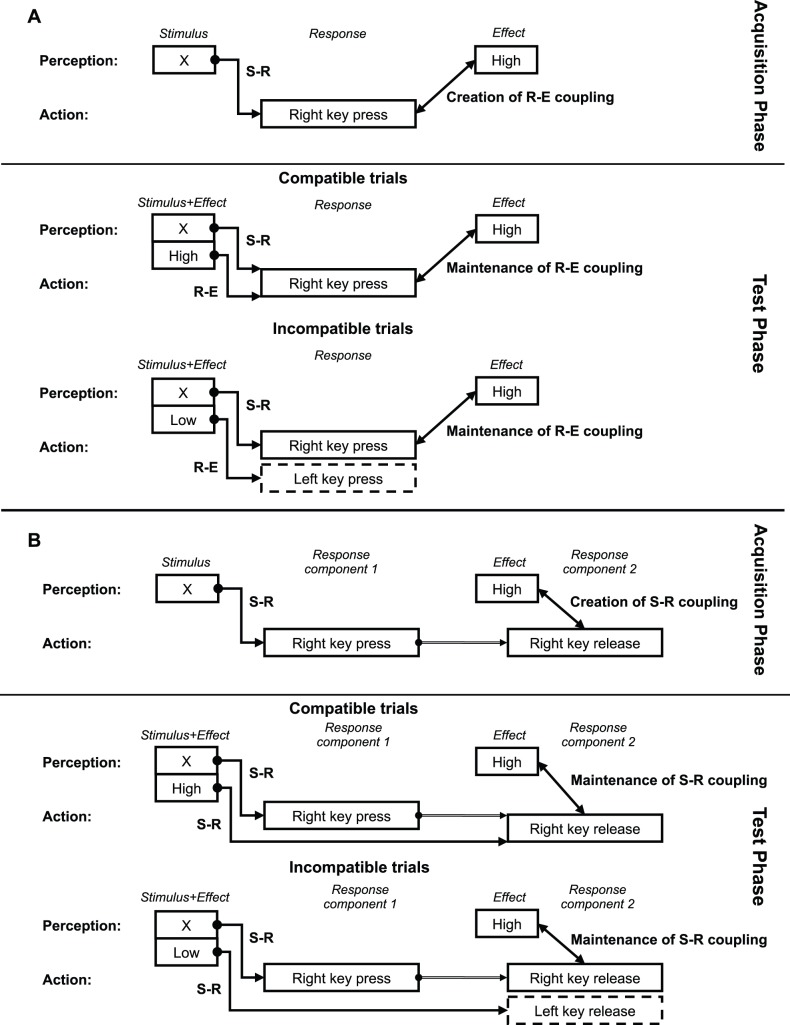
Outline of the two models. (**A**) Outline of the two-step model for the ideomotor principle (see e.g. [21]). Response-effect (R-E) couplings are established between key press and tone in the acquisition phase. This results in response facilitation in the test phase when at stimulus presentation the matching tone (with respect to this stimulus) is sounded, but not when the other tone is sounded. (**B**) Outline of the alternative model based on stimulus-response (S-R) couplings established between Stimulus and Response component 1 and between Effect and Response component 2. The difference in response latencies between compatible and incompatible trials for both movements is accounted for by cooperation and competition mechanisms between the two response-selection processes (triggered by the S-R couplings). Importantly, the two response components are to be understood as an integrate part of the same ongoing response action. This schematic merely temporally separates them along the time line of the task.

**Figure 2:**
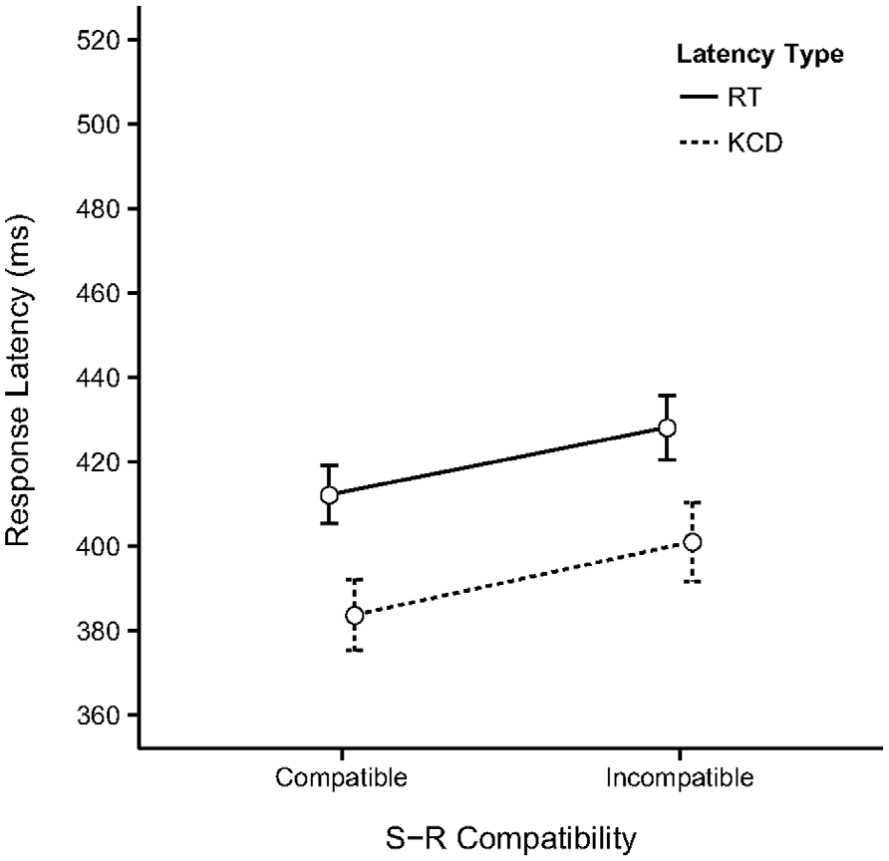
Results of Experiment 1. The observed means (with 95% CI) for the two types of response latency measured in Experiment 1 as a function of S-R Compatibility of the trials. The values of the means, confidence intervals and size of the Compatibility Effect (CE) are reported in Table S1.

In an influential series of studies, Hommel and coworkers ([20]–[22]; see also [23], [24]) use this design in the context of a choice reaction task. The general experimental procedure is as follows (see Figure 1A): In the acquisition phase, participants have to press one key after seeing a certain stimulus and another key after seeing a different stimulus (e.g., ‘Right key’ following ‘X’ and ‘Left key’ following ‘O’). Immediately after each key press a specific tone is sounded, with a different pitch for each key (e.g., ‘High pitch’ following ‘Right key’ and ‘Low pitch’ following ‘Left key’). In the subsequent test phase, the tone is sounded already at stimulus presentation. Of the four possible stimulus+tone combinations, two are compatible in the sense that both should trigger the same response (i.e., a specific key press), and two are incompatible because of their conflicting response associations. The results seem to be in accordance with the two-step model described above: Response times in compatible trials are lower than those in incompatible trials (for an example of the results of such an experiment, see the solid [RT] line in Figure 2). That is, response selection in the test phase appears to be prompted by the response-effect associations that are formed in the acquisition phase between each specific key press (i.e., ‘Right key’ and ‘Left key’) and a specific tone (i.e., ‘High pitch’ and ‘Low pitch’, respectively).

In the present study we started from the same basic design. However, as revealed by a careful examination of the event sequence *within* each trial of the task, each response action in a simple or choice reaction task consists of two subsequent but connected movement parts: After having pressed the key, participants always spontaneously release the key again before the start of the next trial, without being explicitly instructed to do so. Importantly, the key-press movement and key-release movement are an integrate part of the same ongoing action, although they take place at subsequent instances in time and (partly) involve the recruitment of different motor subsystems. Previous studies have neglected the latter part of the action in reaction-time tasks, and, consequently, not much is known about the role of the key-release movement in this context. One exception is a recent study demonstrating that key-press and key-release response latencies are uncorrelated and that both movement parts can be manipulated independently of each other [25]. This suggests that, although both movements are clearly part of the same response action, separate control mechanisms might be at work.

Following on this ‘two-component’ response notion, in Experiment 1 we measured key contact duration in addition to response time in a replication of Hommel’s study [22] (Experiment 3 therein). Starting immediately after the key has been pressed, *key contact duration* measures how long it takes until the participant releases the key again. This provides an indication of how long it takes to select the appropriate second movement-part of the response action. Although we expect to find similar results as discussed above for the response times, no specific hypotheses can be derived from the two-step model with respect to the key-release times. However, if key release is merely a reflexive and arid retraction movement following key press, unrelated to the other aspects of the task (as might be taken from the absence of key release in the two-step model), there is no reason to expect any difference in key contact duration between the compatible trials and the incompatible trials. In that case, motor codes for the key-release movement would be available equally fast after the key is pressed. However, if we find a difference in key contact duration between these trial types, we must consider an alternative explanation. This explanation should incorporate the key-release movement as an integral and relevant component of the complete response action, and of the entire task, as suggested in this paper.

## Experiment 1

### Method

#### Ethics statement

Participation in this study was voluntary and with written informed consent. The study was approved by the local ethics committee of the Behavioural Science Institute of the Radboud University Nijmegen.

#### Participants

Twenty-five undergraduate students with a mean age of 19.8 years (*SD* = 2.1 years; *range* = 17–27 years) participated in the experiment, for which they were rewarded 5 euros or course credits. All participants were naïve to the purpose of the experiment, were right-handed and had normal or corrected-to-normal vision.

#### Experimental design, materials, and procedure

In Experiment 1 we used the same set up as was used in Experiment 3 of Hommel’ study [22]. Participants were seated at a table in front of a 15-inch monitor, with their hands on a buttonbox. First, participants performed 200 acquisition trials, each consisting of the following sequence of events: At the start a fixation asterisk (*) was shown for 500 ms, which was followed by the stimulus presentation after 150 ms. The stimulus was either the letter ‘X’ or the letter ‘O’, appearing for 150 ms, at random, 100 of each in total. Participants had to respond to this stimulus as quickly as possible by pressing the corresponding right or left key. As an effect following this key press, a specific tone was sounded for 100 ms, with a pitch of either 300 Hz or 600 Hz. Participants were told to ignore the tones.

In the subsequent test phase, another 80 trials were performed. These trials were identical to the trials in the acquisition phase, except that now one of the tones was sounded simultaneous with presentation of the letter. In half of the trials, letter and tone matched with respect to the response that was required or that seemed to have caused it in the acquisition phase, respectively. These were the compatible trials (C trials). In the incompatible trials (IC trials), stimulus and tone corresponded to different responses in this respect. In the test phase, the tone was still sounded after the key press, to maintain the coupling between action and effect. All possible stimulus (letter; X/O) – response (key; right/left) – effect (tone; high/low) combinations were counterbalanced across participants.

Two types of response latency were measured: Response time (RT) was defined as the time interval between stimulus presentation and pressing of the response key. Key contact duration (KCD) was defined as the time interval between pressing the response key and releasing that key again.

#### Data analysis: General strategy

We followed a response latency analysis strategy that involved fitting linear mixed effect models to transformed latency data with moderate a-priori trimming (see [26] and [27]). The procedure was the same for all experiments reported in this paper: 1. Find a proper scale for the data (log transform); 2. Remove data points that are physically, or by design impossible (responses faster than 50 ms and slower than 1000 ms were removed); 3. Check the distribution for each participant with a Shapiro-Wilk test [27]. The proportion participants whose latency distribution needed outlier removal in each experiment did not exceed 15% of each sample. The outliers were detected based on a distribution fit of the data (e.g. [28]). Up to a maximum of 5 data points marked as outliers on the left side of the distribution and 5 on the right side were removed; 4. Analyze and exclude erroneous responses. Note that Hommel and coworkers [20]–[22] do not exclude error responses before analysis, whereas others [23], [24] do exclude errors; 5. The modeling strategy was the same for all experiments: A linear mixed effect model was fitted by maximum likelihood with Latency as the dependent variable. The fixed effects in the model were: S-R Compatibility with the levels Compatible (C) and Incompatible (IC) indicating the trial type; Latency Type with the levels Reaction Time (RT) and Key Contact Duration (KCD) indicating the response type under analysis; the interaction between S-R Compatibility and Latency Type. The factors were defined such that the fixed Intercept coincided with Reaction Time of the Compatible kind. The random effects in the model were trials nested within participants; random intercepts and slopes were estimated for each participant. The predictions deduced from our hypotheses in all three experiments were about the presence or absence of a Compatibility Effect (CE) associated with a response type (key-press or key-release). For each set of experiments we conducted a model based planned comparison (i.e. simultaneous tests for general linear hypotheses [29]) of the CE (Latency of IC trials>Latency of C trials) for the two types of measured response latency.

The data were analyzed in R [30] using the *extremevalues* package [31] for outlier detection, the *nlme* package [32] for fitting the linear mixed effects model and the *multcomp* package [33] to conduct the multiple comparisons after *lme* modeling. Appendix S1 is a.zip archive that contains the raw data and annotated R scripts that were used to obtain all the results reported in this paper.

### Results

The Percentage Error (PE) was 2.0% of valid trials in Experiment 1 and was analyzed as a function of S-R Compatibility of the trials (e.g. [22], [23]). There was no significant difference in the errors made on valid compatible and incompatible trials (1.9% vs. 2.1%; *t*(24) = −0.20, *p = *0.846 ). The error trials were discarded and further analyses were conducted on 97.0% of the raw data (see table S1 for a breakdown of PE and valid cases into trial type).

Figure 2 displays the observed means (with 95% CI) for the two types of response latency measured in the experiment as a function of S-R Compatibility. The values of the means and confidence intervals are reported in Table S1. The results of the linear mixed model fit (for details see Table S2) reveal there is a significant main effect of S-R Compatibility (Estimate = 0.03, *SE* = .01, *t*(3826) = 2.82, Pr(>|*t*|) = 0.005) indicating IC trials elicit slower responses than C trials. There was also a significant main effect of response type (Estimate = −0.10, *SE* = 0.01, *t*(3826) = −8.88, Pr(>|*t*|) = 0.001) points to faster responses associated with key-releases (KCD latencies<RT latencies). The interaction between S-R Compatibility and response type was not significant (see Table S2 for details). A comparison of the CE for each response type separately was made using a simultaneous test for general linear hypotheses. This revealed a significant effect for both RT (RT_IC-C_ = 0.032, *SE* = 0.011, *z* = 2.82, Pr(>*z*) = 0.005) and KCD (KCD_IC-C_ = 0.036, *SE* = 0.011, *z* = 3.15, Pr(>*z*) = 0.002; Note that we round to three digits when reporting the small difference score estimates due to the log scale.). The reported p-values were adjusted for multiple comparisons using the Bonferroni method.

Summarizing, these results appear to indicate the effect of trial compatibility occurs for the key-release movement as well as for the key-press movement. In fact, the CE based on the predicted latencies is 14–15 ms for both response types (Table S1).

### Discussion

Why does this effect occur for the key release in the test phase? Since there was no external sensory effect following key release in the acquisition phase, no response-effect (R-E) coupling could have been formed with this movement. This means that the difference in key contact duration between the compatible and incompatible trials of the test phase cannot be explained within the two-step model for the ideomotor principle.

However, notice the temporal contiguity between the sounding tone and the key-release movement in each trial of the task. It is because of these repeated co-occurrences that stimulus-response (S-R) couplings were established between them. To be more precise, each of the two release movements, that is, release of the right key or of the left key, was specifically coupled to one of the two tones, that is, either to the high pitch tone or to the low pitch tone. Building on this observation, we propose a more parsimonious model with S-R couplings rather than R-E couplings, to account the results of the effect-priming studies (see Figure 1B). As will be demonstrated below, this model can explain the compatibility effects for both action components.

In order to explain the compatibility effect in effect-priming studies in terms of S-R couplings we depart from the same source upon which the two-step model and the common-coding framework are built; William James’ description of the ideomotor principle: “every representation of a movement awakens in some degree the actual movement which is the object; and awakens it in a maximum degree whenever it is not kept from so doing by an antagonistic representation present simultaneously to the mind” [1]. Incompatible trials are such cases in which the actual movement is not activated “in a maximum degree” due to simultaneous presentation of stimuli that are coupled to antagonistic (aspects of) movements. Compatible trials are cases in which it is assumed that “every representation of a movement awakens in some degree the actual movement”, refers to some additive or cooperative effect in movement activation by different stimuli that are coupled to an aspect the actual movements share.

We make the assumption that conflicting cues about the laterality of a movement to be executed by an index finger give rise to a higher degree of antagonism or competition than conflicting cues about the vertical direction (i.e. press or release) of a movement when laterality cues are not in conflict, comparable to the Simon effect. For incompatible trials the stimulus-tone presentation will result in increased response latency for the key-press and key-release movements because the tone will activate a movement that differs in laterality from the movement the stimulus activates. As the key is pressed and the effect-tone is sounded, both events activate the key-release action for the finger that performed the key-press action. This will compete with the movement activation elicited by the tone presented at the beginning of the trial that activated the key-release action of opposite laterality (see Figure 1B). In compatible trials the stimulus-tone combination is always in accordance with the laterality of the movements that are activated and after the key-press action is performed, the effect tone, the key-press movement and the tone presented at the beginning of the trial are not in conflict to activate the key-release action.

Note that this mechanism is essentially the same as the one proposed by the two-step model. Both models accommodate for the formation of contiguity associations between various perception and action components in the task. They differ only in the theoretical units used to explain the compatibility effect: S-R couplings between observables that can be precisely defined in the physical environment of the brain versus R-E couplings between abstract representations of sensorimotor codes stored in the brain.

To elaborate, these associations, which do the explanatory work in the models and account for the empirical findings, have a fundamentally different ontological status. In the S-R model, one-way couplings are formed between stimuli and subsequent responses as simple conditioned associations of the stimulus and response context, whereas in the two-step model bi-directional couplings are created between encoded representations of aspects of responses and subsequent effects. It is our claim that the compatibility effect found in effect priming studies does not necessarily imply such R-E couplings exist, nor that James’ ideomotor principle is actually at work in these studies. The compatibility effect can completely and elegantly be understood within a framework of S-R couplings as suggested by Watson’s [34] or perhaps even by means of Pavlov’s classical conditioning [35].

The subsequent experiments explicitly tested the alternative model for the ‘priming’ effect introduced above. In Experiment 2A and 2B the two movement-components of the response action were isolated (key-press only in Experiment 2A and key-release only in Experiment 2B). According to the two-step model this decomposition should have no influence on the effect. Consequently, a null-result in these experiments would falsify the two-step model in favor of the alternative model. In Experiment 3A and 3B the acquisition phase consisted of two response-components again, but now no imperative exogenous stimulus was presented in the task. This part replicated Elsner and Hommel’s study [21] (Experiment 1 therein; see also [23]), which is an improved version of the earlier procedure used in Experiment 1 (see [22]). In the test phase however, participants again only performed one response-component (key-press only in Experiment 3A and key-release only in Experiment 3B), where the earlier effect now served as stimulus. Differences in the pattern of response latencies between the two versions of the experiment will demonstrate whether the effect in the acquisition phase is either coupled to the first (key-press) movement, as accounted for and explained by the two-step model, or the second (key-release) movement, as accounted for and explained by the alternative model.

## Experiment 2A and 2B

To verify whether the S-R model proposed above indeed accurately and completely captures the mechanisms that lead to the results found in effect-priming choice reaction tasks, we performed a second set of experiments. These were designed to specifically test the actual formation of R-E couplings in the acquisition phase. Experiment 2A and 2B again followed the general procedure described earlier, but ruled out the possibility of forming a second S-R coupling in the task by explicitly eliminating the second movement. This was done in two different ways: Either by asking participants to keep the key pressed after responding to the stimulus (i.e. not releasing again; Experiment 2A) or by asking them to perform a key-release action as the single-component response (Experiment 2B).

### Method

#### Ethics statement

Participation in this study was voluntary and with written informed consent. The study was approved by the local ethics committee of the Behavioural Science Institute of the Radboud University Nijmegen.

#### Participants

A total of 52 undergraduate students with a mean age of 20.5 years (*SD* = 2.3 years; *range* = 18–28 years) participated in the experiment, for which they were rewarded 5 euros or course credits. All participants were naïve to the purpose of the experiment, were right-handed and had normal or corrected-to-normal vision.

#### Experimental design, materials, and procedure

The main design (i.e., letter stimuli, effect tones, timing and duration of events, and number of trials in both phases of the task) was identical to Experiment 1, except for the required response. Half of the participants (*N* = 26) were assigned to the key-press only variation (Experiment 2A), where at the start of each trial in the acquisition phase they were instructed by a message on the screen, to keep their index fingers over the keys. After the fixation asterisk, upon stimulus presentation, they had to press the appropriate key and keep it pressed. This key press triggered the sounding of the corresponding tone. Thousand milliseconds after this, a message appeared on the screen instructing participants to release the key and prepare for the next trial by holding both fingers over the keys again. The other half of the participants (*N* = 26) performed the key-release only variation of the (Experiment 2B), in which they were instructed by a message on the screen to press down both keys with their index fingers before the start of the trial. Now, upon stimulus presentation, they had to release (only) the appropriate key without pressing it again. In this variation, key release triggered the tone. Before the start of each new trial they were asked to prepare for the next stimulus and press both keys again.

Response time and key contact duration were defined as the time interval between stimulus presentation (response cue) and key press or key release, respectively. Note that key contact duration plays a different role in Experiment 2 compared to Experiment 1.

Similar to Experiment 1, in the test phase of both variations, letter and tone were presented together at the beginning of each trial again. The rest of the procedure was the same as in the acquisition phase.

### Results and Discussion

The PE was 4.1% of valid trials in Experiment 2A (RT) and 5.8% in Experiment 2B (KCD). There were no significant differences in the errors made on valid C and IC trials (RT: 3.1% vs. 5.2%, *t*(25) = −2.04, *p = *0.052; KCD: 4.9% vs. 6.6%, *t*(25) = −1.44, *p = *0.162). The error trials were discarded and further analyses were conducted on 95.8% of the measured RT trials and 93.8% of the KCD trials (see Table S1 for a breakdown of PE and valid cases into Latency Type and S-R Compatibility of trials).

**Figure 3:**
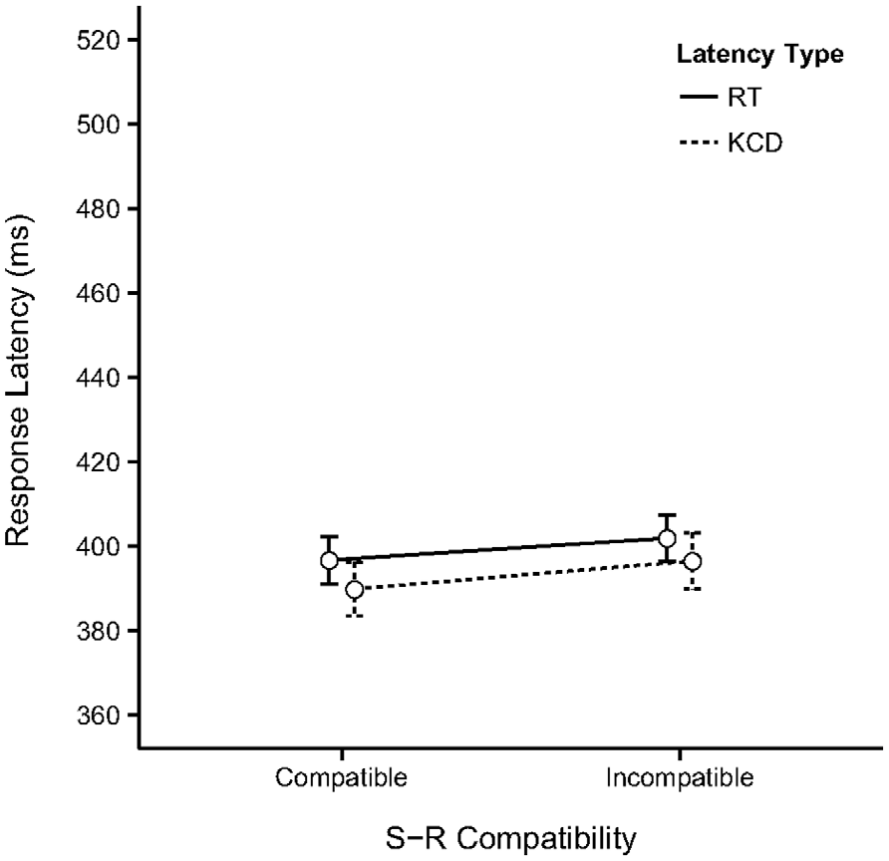
Results of the test phase of the two single-movement response variations of Experiment 2. The observed means (with 95% CI) for the two types of response latency measured in Experiments 2A and 2B as a function of S-R Compatibility of the trials. The values of the means, confidence intervals and size of the Compatibility Effect (CE) are reported in Table S1. In Experiment 2A participants had to press and hold down the correct key upon stimulus presentation, in Experiment 2B participants had to release the correct key upon stimulus presentation.

Figure 3 displays the observed means (with 95% CI) for the two types of response latency measured in the experiment as a function of S-R Compatibility. The values of the means and confidence intervals are reported in Table S1. The result of the linear mixed model fit show there are no significant main or interaction effects in the model (see Table S2 for details). The predicted difference between IC and C trials does appear to be in the direction of a CE: CE_RT = _5 ms, CE_KCD = _6 ms. Our prediction for this experiment was the absence of a CE, but we can of course only show it is statistically unlikely there is a compatibility effect. A testable hypothesis that could add some more credibility to our prediction would be that CE for each response type in Experiment 2 should at least be significantly smaller than the CE observed in Experiment 1 (H_1_: CE_EXP2_< CE_EXP1_). The estimated CEs obtained from the previously reported simultaneous test of trial compatibility in the linear effects model fitted to the data of Experiment 1 were used as critical values to the CEs predicted by the model fitted to the data of experiment 2. Results show the compatibility effects for RT and KCD were significantly smaller in Experiment 2 compared to Experiment 1 (RT_IC-C_EXP1_ = 0.032, RT_IC-C_EXP2_ = 0.013, *SE* = 0.009, *z* = −2.14, Pr(>*z*) = 0.03); KCD_ IC-C_EXP1_ = 0.036, KCD_IC-C_EXP2_ = 0.018, *SE* = 0.009, *z* = −2.03, Pr(>*z*) = 0.04. The reported p-values were adjusted for multiple comparisons using the Bonferroni method). The estimated upper bound of the family-wise 95% CI for the difference between IC and C trials was 0.031 and 0.035 for RT and KCD respectively.

Results of both experiments are clear: There was no longer any evidence of facilitation in action selection caused by effect priming. More specifically, the difference between the compatible and incompatible response latencies in the test phase was negligible in both experiments.

Any null-result, even when predicted, has obvious interpretation difficulties. To provide additional evidence that it is unlikely that the priming effect was present we compared the size of the compatibility effects found in Experiment 1 with those found in Experiments 2A and 2B. The compatibility effect for each response type in Experiment 2 was smaller than the CE found in Experiment 1. The cross-experiment comparison, in addition to the non-significant estimated effect sizes in the linear mixed model make it highly unlikely that there was a priming effect present in Experiment 2A and 2B.

## Experiment 3A and 3B

The results of Experiment 2A and 2B might be explained alternatively by referring to the difference that exists between the sensorimotor (stimulus-based) mode and the ideomotor (intention-based) mode of ideomotor learning. The distinction between these two modes was recently made by Herwig, Prinz, and Waszak ([23]; see also [24]). Their study demonstrated that participants only form action-effect associations when they are working in the intention-based action mode. That is, whenever the action to be performed is selected endogenously rather than exogenously. Within that framework the lack of forming R-E couplings in both Experiment 2A and 2B would be attributed to the fact that the acquisition phase of both versions was performed in the sensorimotor mode rather than the ideomotor mode.

Another possible shortcoming of the design of the previous experiments is that strictly speaking no direct predictions with respect to possible differences in response-effect associations between one-movement and two-movement actions can be derived from the two-step model. The model simply does not incorporate an analysis of different movement components in its description of the underlying mechanisms. However, it does not specifically proscribe this distinction either. So, on the one hand, there is nothing in the two-step model that would exclude the one-movement actions from the domain of ideomotor learning. But, on the other hand, it might still be opposed that a deconstruction of the action, and the resulting separation of the movement components, as was done in Experiment 2A and 2B, affects the mechanism of ideomotor learning to the extent that it impedes with the formation of R-E couplings. Although such a disruption cannot be explained within the two-step model as yet, it nevertheless may weaken our conclusions based on the one-movement acquisition phase.

**Figure 4:**
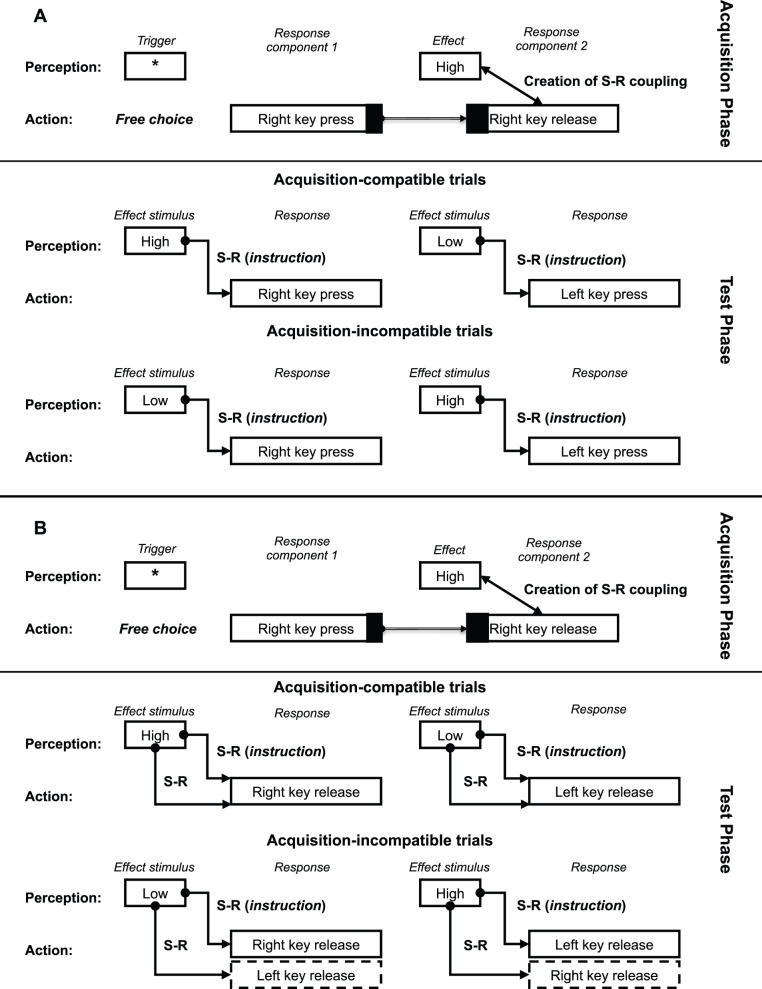
Setup of Experiment 3A and Experiment 3B in terms of the S-R model. The acquisition phase of both versions of the experiment was identical, consisting of two response components and without an imperative stimulus, such that they were performed in the ideomotor mode (for more details see text). (**A**) In the test phase of the key-press only version (Experiment 3A), response selection is not facilitated by the S-R coupling between Effect (tone) and Response component 2 (key release). (**B**) In the test phase of the key-release only version (Experiment 3B), this S-R coupling does influence response selection, leading to differences in response latencies between the compatible and incompatible conditions.

These concerns give rise to the following two alterations in the design of the previous experiments (see Figure 4): First, in the following set of experiments no imperative exogenous stimulus was presented in the acquisition phase, in order to assure that this phase was performed in the ideomotor mode [23]. In the test phase, the effect served as the stimulus in a forced-choice reaction task. Second, the acquisition phase contained a two-component response. That is, participants could release the key at will after pressing it, without being explicitly instructed about this second movement. Contrary, in the test phase, a one-movement response was required from the participants. The acquisition phase of the following set of experiments was similar to that of Experiment 1 in Elsner and Hommel’s study [21], whereas the test phase only differed with respect to the number of response components. Together these alterations also make sure that the latest version of the response-effect paradigm was used in this critical last experiment.

This design not only addressed the two critical notes discussed above, it also resulted in a clear distinction between the two-step model and the stimulus-response model introduced here: The two-step model predicts R-E couplings that link the first response-component (key press) to the subsequent effect (tone), whereas the S-R model predicts S-R couplings between the effect (tone) and the subsequent second response-component (key release). These predictions translate into sharply distinct patterns of response latencies in the one-movement test phase: The difference between the acquisition-compatible and acquisition-incompatible condition is either present in the key-press only version (Experiment 3A; as predicted by the two-step model) or in the key-release only version (Experiment 3B; as predicted by the S-R model). The mechanisms according to the S-R model are visualized in Figure 4.

### Method

#### Ethics statement

Participation in this study was voluntary and with written informed consent. The study was approved by the local ethics committee of the Behavioural Science Institute of the Radboud University Nijmegen.

#### Participants

In these experiments a total of 120 undergraduate students participated. The mean age of this group was 21.1 years (*SD* = 2.5 years; *range* = 18–31 years). Participants were naïve to the purpose of the experiment, were right-handed and had normal or corrected-to-normal vision. They received 5 euros or course credits for their participation.

### Experimental Design, Materials, and Procedure

#### Acquisition phase

In the 200 trials of the acquisition phase, participants pressed one of the keys at will, as quickly as they could, following a non-specific trigger. No imperative exogenous stimulus was presented, making this a free-choice reaction task instead of a forced-choice reaction task like Experiment 1 and Experiment 2. Participants were instructed to randomly select which key to press, but to keep the total number of left and right key presses about equal. After 100 trials subjects were informed how many times they had pressed the left key and the right key. No instructions were given with respect to key release. Pressing a key triggered one of two different tones (300 Hz or 600 Hz) to be sounded for 100 ms, where each tone specifically followed either a right-key press or a left-key press. Response (Key) –effect (tone) combinations were counterbalanced across participants.

#### Test phase

In the test phase, only a single-movement response was required. As in Experiment 2, there were two variations (see Figure 4): Experiment 3A was the key-press only variation and Experiment 3B was the key-release only variation. At the start of this phase, participants were instructed to perform a stimulus-response experiment, in which the two effect tones were used as target stimulus, specifically coupled to a key press (Experiment 3A) or a key release (Experiment 3B). They had to respond to the stimulus (either ‘high’ or ‘low’ pitch tone) as quickly as possible, with either the left key or the right key. In of both experiments there were two compatible conditions: One subgroup performed an acquisition-compatible stimulus-response combination. These participants had to respond to the stimulus (i.e. effect tone) by pressing or releasing the key that was always paired with this effect tone in the acquisition phase. The other subgroup performed an acquisition-incompatible stimulus-response combination. These participants had to respond to the stimulus (i.e. effect tone) by pressing or releasing the opposite key, with respect to the link in the acquisition phase. The specific stimulus (tone) – response (key) combination was instructed before the start of the test phase. Participants were assigned randomly to the conditions and variations of the experiment, such that each of the four combinations included 30 participants. The test phase consisted of 100 trials.

Response time and key contact duration were defined similar to Experiment 2.

### Results and Discussion

During the acquisition phase of the key-press only experiment (3A), participants pressed the right key 50.3% of the time and the left key 49.6% of the time and 0.1% were missed or invalid trials. For the key-release only experiment (3B) the key release percentages were 49.5% for the left key, 50.2% for the right key and 0.3% were missed or invalid trials.

The PE was 2.8% of valid trials in the test phase of Experiment 3A (RT) and 6.4% in Experiment 3B (KCD). As was the case in Experiment 2, the key-release only experiments elicited more erroneous responses than the key-press only experiments did. However, a two sample t-test with Welch adjustment of the degrees of freedom was conducted for each latency type showed no significant differences in the errors made on valid C and IC trials (RT: 3.0% vs. 2.7%, *t*(56.0) = 0.54, Pr(>|*t*|)* = *0.588; KCD: 6.3% vs. 6.5%, *t*(57.7) = −0.06, Pr(>|*t*|) = 0.954). The error trials were discarded and further analyses were conducted on 97.0% of the measured RT trials and 93.1% of the KCD trials (see table S1 for a breakdown of PE and valid cases into Latency Type and S-R Compatibility of trials).

**Figure 5:**
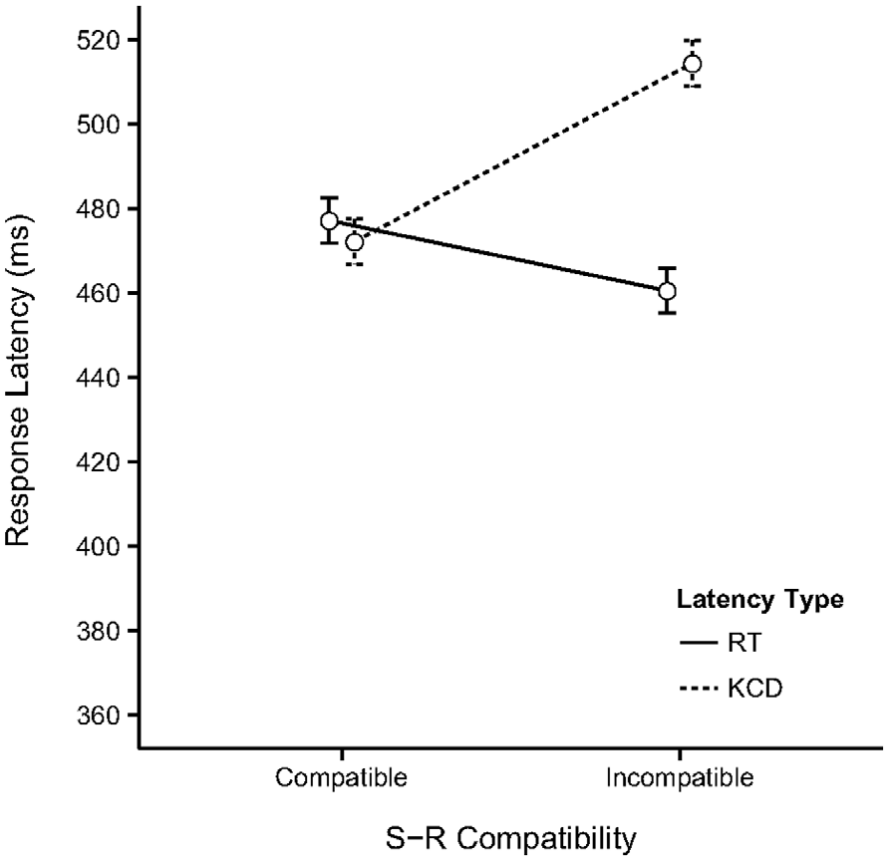
Results of the test phase of Experiments 3A and Experiment 3B in one graph. The observed means (with 95% CI) for the two types of response latency measured in Experiments 3A and 3B as a function of S-R Compatibility of the trials. The values of the means, confidence intervals and size of the Compatibility Effect (CE) are reported in Table S1. Experiment 3A was the key-press only version (RT) and Experiment 3B the key-release only (KCD) version. In these experiments, S-R Compatibility was measured between subjects.

Figure 5 displays the observed means (with 95% CI) for the two types of response latency measured in the experiment as a function of S-R Compatibility. The values of the means and confidence intervals are reported in Table S1. The result of the linear mixed model fit in Table S2 shows that there are no significant main effects, but the interaction effect is significant (Estimate = 0.137, *SE* = 0.063, *t*(116) = 2.16, Pr(>|*t*|) = 0.033). A simultaneous test for general linear hypotheses showed the compatibility effect was only significant for KCD (RT_C-IC_ = −0.044, *SE* = 0.044, *z* = 0.98, Pr(<z) = 1.00); KCD_C-IC_ = −0.095, *SE* = 0.044, *z* = −2.14, Pr(<*z*) = 0.032. The reported p-values were adjusted for multiple comparisons using the Bonferroni method).

The two experiments revealed a clearly distinct set of results: The average response times in the acquisition compatible subgroup of Experiment 3A (key-press only variation) did not significantly differ from the acquisition incompatible subgroup. On the other hand for key contact duration in Experiment 3B (key-release only variation), there was a significant compatibility effect. These results are in accordance with the prediction based on the S-R model and are inconsistent with the predictions of the two-step R-E model.

### Conclusions

The incentive of the present study was to investigate ideomotor action control, starting from a more elaborate than usual conception of the action that participants perform in a reaction-time task. Incorporating not only the (first) key-press response-component but also the (second) key-release response-component into the task analysis leads to the proposition of a twofold stimulus-response model. This model was motivated by the results of Experiment 1 and supported by those of Experiment 2A and 2B. Finally, our S-R model was corroborated in a direct test against the two-step R-E model in Experiment 3A and 3B, thereby falsifying the latter model. More specifically: In Experiment 1 the earlier findings of key-press response facilitation were replicated and extended to the key-release movement. Contrary, each of the action components loses the ability to be influenced by effect priming when separated, as was done in Experiment 2A and 2B. Moreover, as was demonstrated unambiguously in Experiment 3A and 3B, using the most advanced and latest experimental setup in this field, the effect did not influence the (preceding) key-press movement but did influence the (succeeding) key-release movement.

Together our results challenge the belief that the compatibility effect found in movement selection within the effect priming paradigm is caused by previously established couplings between these movements and their subsequent effects, as has been suggested by others (e.g. [20]–[24]). As a result, given that it is unclear how to incorporate the present findings into the two-step R-E model, it is difficult to maintain this model as a complete and consistent description of the mechanisms underlying the ideomotor principle. It is important to stress that this conclusion is independent of whether the S-R based explanation presented here as an alternative model might be proven incorrect by future studies.

A possible objection to this study might be that the setup that was chosen in the last two experiments, particularly the single-movement actions, renders the two-step R-E model inapplicable. First of all, as said before, there is nothing in the two-step R-E model that suggests that single-movement actions cannot be coupled to effects. This would in fact imply the nonexistence of an associative mechanism for this type of actions, and, in that case, the common-coding framework would face a serious generalization problem. Besides, an alleged inappropriateness of single-movement actions for associations can only explain the null-results of Experiment 2. Since the acquisition phase of Experiment 3 involved the canonical (i.e. two-movement) actions, no obstacle for forming associations was presented there. And clearly associations were formed in this experiment, but the outcome was exactly as predicted by the alternative S-R model. So irrespective of whether responses (being of the single- or double-movement type) and effects can actually be coupled –and there is nothing that suggests they cannot–, we can be certain that only the S-R couplings played a role in the service of response selection. This conclusion is supported by the present results, particularly those of Experiment 3, providing evidence that response-effect associations are not straightforwardly functional in anticipatory action control.

It might still be objected that the single-movement actions used in Experiment 2 and in the test phase of Experiment 3 are rather atypical, and therefore perhaps impractical or difficult to perform for participants. Because of this, it might be argued that action control places relatively large demands on cognitive and attentional resources, which leads to a reduced role of action-effect associations. However, both types of single-movement responses are part of the standard action repertoire that most modern individuals use on a daily basis. For instance, human-computer interactions, such as scrolling texts in electronic documents or websites using touch-pad, mouse or keyboard, require multiple variations of extended/continued pressing movements or releasing movements of the fingers. It was the case that experiment 3 elicited more error responses than experiments 1 and 2. This was likely due to the ‘sudden’ change in response action instruction after the regular response action during the acquisition phase. The error responses did not confound our results however, as they did not vary systematically with trial compatibility. There is thus no reason to expect that these particular actions would pose any additional control constraints, compared to the two-movement actions of Experiment 1 and the acquisition phase of Experiment 3. This conclusion is supported by the observation that the response times of the two-movement and one-movement response actions in experiment 1 and 2 respectively are comparable after the one-movement actions have been sufficiently practiced in the acquisition phase (see Figure 2 and 3). There was no practice opportunity in experiment 3, explaining the overall higher response latencies (see Figure 5).

On a more general note, the present study clearly emphasizes the limited and well-defined status that simple contiguity associations have in the context of human action control. Their existence and function seems to be restricted to the realm of reactive and reflexive control of behavior. On the other hand, mechanisms of a more complex organization are likelier to be involved in the pro-active, prospective and voluntary part of our behavior (see also [36]). In conclusion, we propose that a better understanding of how the action system becomes future oriented and goal directed will have to come from a more complete definition and measurement of action, as suggested here, focusing on how smaller movement units are part of the larger context of the action. This notion is at least partly recognized within the common-coding framework and the theory of event coding, when referring to the complexity of action control in real life versus that in a laboratory experiment: “And yet, most models of action control seem to take this highly artificial stimulus-response situation so serious that they use it as a template for voluntary action in general.” [37] Curiously, this insight does not lead to any significant changes in the way the theory of event coding investigates and accounts for action control.

Importantly, the alternative S-R model we presented in no way disproves or challenges the truth status of the ideomotor principle [1]. The model merely demonstrates that effect-primed choice reaction tasks are not about ideomotor theory, and vice versa, that the ideomotor principle is not about effect priming.

Finally, the results presented here might have serious implications for priming studies in other fields of psychology as well (see [38]). Perhaps the rationale and working model (based on S-R associations) could play a fruitful role in devising novel experimental procedures and designs to test the nature of priming effects more generally. We recommend critical evaluation of the viability of the ontology assumed for the associations between prime and primed response by a detailed task analysis.

## Supporting Information

Table S1 (PDF)

Table S2 (PDF)

Appendix S1 (ZIP)
