# Supplementary material for: The Case of Watson vs. James: Effect-Priming Studies Do Not Support Ideomotor Theory
Source: PLoS One. 2013 Jan 22;8(1):e54094. doi: 10.1371/journal.pone.0054094 (PMC3551954; doi:10.1371/journal.pone.0054094)
Supplement: Table S1 — (PDF) [file pone.0054094.s001.pdf]

Table S1. Summary of the results grouped by Experiment (1, 2AB, 3AB), Latency Type (Response Time, Key Contact Duration) and S-R Compatibility (Compatible, Incompatible).

Experiment	N	Latency Type	S-R Comp.	PE	Valid Cases	Observed Latency (95% CI)	Observed CE	Predicted Latency (95% CI)	Predicted CE
1	25	RT	C	1.9%	97.0%	412 (405-419)	16	407 (402-412)	14*
	|	RT	IC	2.0%	97.1%	428 (420-436)		421 (416-426)	
	|	KCD	C	|	|	384 (375-392)	17	367 (363-372)	15*
	|	KCD	IC	|	|	401 (392-410)		382 (377-386)	
2A	26	RT	C	3.1%	97.1%	397 (391-402)	5	390 (387-392)	5
	|	RT	IC	5.2%	94.5%	402 (396-407)		395 (393-398)	
2B	26	KCD	C	4.9%	94.8%	390 (383-396)	6	381 (377-384)	6
	|	KCD	IC	6.6%	92.8%	396 (390-403)		387 (384-391)	
3A	30	RT	C	3.0%	96.5%	477 (472-482)	-17	464 (461-467)	-15
	30	RT	IC	2.7%	97.6%	460 (455-466)		449 (446-452)	
3B	30	KCD	C	6.3%	94.0%	472 (467-477)	42	459 (456-462)	42*
	30	KCD	IC	6.5%	92.1%	514 (509-520)		501 (498-504)	

Note: The table shows the sample size N, the percentage errors made (PE), the % of measured observations used in the analyses after outliers and errors were removed (Valid Cases), the Observed Latency (with 95% Confidence Interval), the compatibility effect based on the valid cases (Observed CE), the latency as predicted by the linear mixed effect models (with 95% Confidence Interval) and the compatibility effect based on the linear mixed effect models (Predicted CE). Marked CEs are significant in post-hoc analysis. Notice that RT and KCD were measured within subjects in Experiment 1, whereas in the other experiments they were measured in separate samples.
