# Supplementary material for: The Case of Watson vs. James: Effect-Priming Studies Do Not Support Ideomotor Theory
Source: PLoS One. 2013 Jan 22;8(1):e54094. doi: 10.1371/journal.pone.0054094 (PMC3551954; doi:10.1371/journal.pone.0054094)
Supplement: Table S2 — (PDF) [file pone.0054094.s002.pdf]

Table S2. Summary of the fixed effects of the linear mixed model fitted to the response latencies of Experiment 1, 2AB and 3AB.

	**Experiment 1**			**Experiment 2AB**			**Experiment 3AB**		
Model Parameter	*Estimate*	*t(df)*	*Pr(>|t|)*	*Estimate*	*t(df)*	*Pr(>|t|)*	*Estimate*	*t(df)*	*Pr(>|t|)*
Intercept (RT, Compatible)	6.00 (.04)	159.83 (3826)	<0.001	5.99 (.02)	253.46 (3868)	<0.001	6.13 (.03)	196.24 (10923)	<0.001
S-R Comp. (Incompatible)	0.03 (.01)	2.82 (3826)	<0.01	0.01 (.01)	1.51 (3868)	n.s.	-0.04 (.04)	-0.98 (116)	n.s.
Response Type (KCD)	-0.10 (.01)	-8.88 (3826)	<0.001	-0.05 (.03)	-1.46 (50)	n.s.	-0.02 (.04)	-0.51 (116)	n.s.
S-R Comp. X Response Type	0.00 (.02)	0.23 (3826)	n.s.	0.00 (.01)	0.36 (3868)	n.s	0.14 (.06)	2.21 (116)	<0.05
Observations	N = 3854			N = 3922			N = 11043		
Random Effect Structure	Subjects = 25, Trials = 80			Subjects = 52, Trials = 80			Subjects = 120, Trials = 100		
log-likelihood	-219.68			703.29			342.03		

Note: Each model was fitted on log transformed response latencies. Shown are the Parameter Estimate (with standard error), the t-value (with degrees of freedom) and the significance level of the t-value. Random intercepts and slopes were estimated for Trials within Subjects.
